# Evaluation of Cutaneous Manifestations in Patients With Diabetes Mellitus: Association With Non-alcoholic Fatty Liver Disease and Other Gastrointestinal Complications

**DOI:** 10.7759/cureus.95882

**Published:** 2025-11-01

**Authors:** Dilaram Khan, Junaid Ahmad, Momna Arif, Kainat Khan, Jawad Bari, Farhan Ali, Tajala Fayyaz, Salman Haider, Hanif Ullah Hanfi, Naqeeb Ullah

**Affiliations:** 1 Gastroenterology, Lady Reading Hospital Medical Teaching Institution, Peshawar, PAK; 2 Medicine, Hayatabad Medical Complex, Peshawar, PAK; 3 Acute Internal Medicine, Midland Metropolitan University Hospital, Smethwick, GBR; 4 Internal Medicine, Lady Reading Hospital Medical Teaching Institution, Peshawar, PAK; 5 Medicine, Lady Reading Hospital Medical Teaching Institution, Peshawar, PAK; 6 Internal Medicine, Khyber Teaching Hospital Medical Teaching Institution, Peshawar, PAK; 7 Internal Medicine, Mardan Medical Complex, Mardan, PAK

**Keywords:** dermatologic signs, diabetes mellitus, gastrointestinal diseases, non-alcoholic fatty liver disease, skin diseases

## Abstract

Introduction

Cutaneous manifestations occur in approximately 30-70% of patients with diabetes mellitus (DM) and frequently serve as visible indicators of underlying systemic complications, such as non-alcoholic fatty liver disease (NAFLD) and gastrointestinal (GI) disturbances. Early recognition of these dermatological signs can facilitate timely diagnosis, improve risk stratification, and contribute to more comprehensive metabolic management. Given the shared metabolic pathways among skin, hepatic, and GI abnormalities, exploring their interrelationships may provide insight into the systemic nature of diabetes.

Objective

To evaluate the prevalence and pattern of cutaneous manifestations among diabetic patients and to determine their association with NAFLD and GI complications. We hypothesized that specific cutaneous manifestations may correlate with hepatic and GI involvement in diabetes.

Materials and methods

A cross-sectional analytical study was conducted at Lady Reading Hospital (LRH), Peshawar, Pakistan, over 12 months, enrolling 328 patients with DM. Participants were evaluated for cutaneous manifestations, GI symptoms, and NAFLD confirmed by abdominal ultrasonography. A structured proforma was used to record demographic and clinical parameters, including age, gender, body mass index (BMI), duration of diabetes, and HbA1c. GI symptoms were assessed using a self-designed, structured symptom checklist developed in consultation with gastroenterologists and pretested for clarity and consistency. Data were analyzed using SPSS Statistics, version 26.0 (IBM Corp., Armonk, NY). Associations were determined using the chi-square and Fisher’s exact tests, while multivariate logistic regression was applied to identify independent predictors of NAFLD after adjusting for confounders.

Results

Cutaneous manifestations were identified in 226 (68.9%) participants, with acanthosis nigricans (n = 92; 28.0%) and diabetic dermopathy (n = 84; 25.6%) being the most frequent findings. NAFLD was present in 138 (42.1%) patients and showed a significant association with cutaneous manifestations (108/226 [47.8%] vs. 30/102 [28.3%], p = 0.001). Among GI symptoms, constipation (p = 0.02) and bloating (p = 0.04) were significantly more prevalent in patients with skin changes, whereas diarrhea and nausea/vomiting did not demonstrate significant associations. Multivariate logistic regression identified cutaneous manifestations (OR = 2.05, 95% CI: 1.25-3.35, p = 0.004), BMI ≥ 30 kg/m² (OR = 3.12, 95% CI: 1.88-5.19, p < 0.001), and HbA1c ≥ 8% (OR = 1.67, 95% CI: 1.02-2.74, p = 0.04) as independent predictors of NAFLD.

Conclusion

NAFLD and several GI disturbances are significantly associated with cutaneous manifestations in diabetic patients. These dermatological findings, being non-invasive and easily observable, may serve as valuable clinical indicators for identifying individuals at risk of systemic metabolic dysfunction. Integrating dermatological evaluation into routine diabetes care could support earlier detection, comprehensive management, and targeted prevention of hepatic and GI complications.

## Introduction

Diabetes mellitus (DM) is a long-term metabolic disease marked by elevated blood sugar levels brought on by deficiencies in either the action or secretion of insulin, or both [1]. Increases in the prevalence of the disease have become an epidemic problem in global terms, and both the developed and the developing worlds are witnessing steady growth in its prevalence [2]. According to the International Diabetes Federation (IDF), over 500 million people worldwide are currently living with diabetes, a figure projected to rise sharply in the coming decades [3]. This growing global burden underscores the importance of understanding diabetes-related systemic and cutaneous complications. Many systemic disorders of the various organs, including the heart, kidneys, eyes, intestine, and skin, are related to diabetes [4].

Cutaneous manifestations are one of the most obvious, but at the same time, underestimated symptoms of DM [5]. The skin, an offshoot of systemic health, often indicates some sort of metabolic imbalance [6]. Up to 30-70% of individuals develop skin lesions during the course of their diabetes [7]. All these dermatological changes may develop directly as a result of chronic hyperglycemia, poor microcirculation, neuropathy, immune dysfunction, microangiopathy, and increased oxidative stress, which together contribute to impaired skin integrity and delayed healing [8,9]. The skin diseases that are common with diabetes include diabetic dermopathy, necrobiosis lipoidica, acanthosis nigricans, candidiasis, and bacterial infections [9]. Although some of these conditions might not seem dangerous, they are the precursors to the possible systemic complications and the imbalances in metabolism [10].

DM not only alters cutaneous structures but is also strongly associated with gastrointestinal (GI) complications such as gastroparesis, constipation, diarrhea, non-alcoholic fatty liver disease (NAFLD), and hepatobiliary dysfunction [11]. NAFLD, now the most prevalent chronic liver disease globally, is closely linked with metabolic syndrome, obesity, and insulin resistance [12]. It occurs more frequently in diabetic patients than in the general population and may progress to non-alcoholic steatohepatitis (NASH), cirrhosis, or hepatocellular carcinoma [13]. These GI complications further impair glucose regulation, reduce quality of life, and contribute to diabetes-related morbidity [14]. Despite the frequent occurrence of cutaneous manifestations in diabetes, their potential relationship with hepatic and GI complications, particularly NAFLD, remains underexplored. Understanding these associations may aid in early detection, risk stratification, and comprehensive management of diabetic patients [15-17].

Although cutaneous manifestations are frequently observed in diabetes, their potential relationship with hepatic and GI complications, especially NAFLD, remains underexplored. Understanding these interconnections may enhance early diagnosis, risk stratification, and holistic management of diabetic patients [15-17]. Therefore, this study aims to evaluate the prevalence and types of cutaneous manifestations in diabetic patients and to investigate their association with NAFLD and other GI complications.

## Materials and methods

Study design and setting

This cross-sectional study was carried out at Lady Reading Hospital (LRH), Peshawar, Pakistan, over a 12-month period, from August 2023 to July 2024. The study aimed to evaluate cutaneous manifestations in patients with DM and their association with NAFLD and other GI complications.

Sample size calculation

The sample size was calculated using the standard formula for prevalence studies: $n = \frac{Z^2 \, p \, (1 - p)}{d^2}$​​​​. In the formula, n is the required sample size, Z = 1.96 for a 95% CI, p (expected prevalence) = 0.737, and d is the margin of error. The prevalence of cutaneous manifestations among patients with diabetes has been reported as 73.7% [18]. To ensure accuracy and contextual relevance, we selected p (expected prevalence) = 0.737 and d = 0.05. This brought about at least 298 patients in the sample. Adjusting for a 10% non-response rate, the ultimate required study sample size was 328 patients.

Study population and eligibility criteria

Patients aged 18 years and above with a confirmed diagnosis of type 1 or type 2 DM who were willing to participate and provided informed consent were included in the study, irrespective of gender. Patients with a history of alcohol abuse, chronic liver disease other than NAFLD, systemic autoimmune diseases, or severe dermatological disorders of non-diabetic origin were excluded. Individuals receiving immunosuppressive or cancer chemotherapy, as well as pregnant, breastfeeding, or critically ill patients unable to undergo proper clinical testing, were also excluded.

Data collection

A structured proforma, attached in the appendices, was used to collect data on glycemic control (determined by HbA1c), comorbidities, medication history, demographic information, and the type and duration of diabetes. A thorough dermatological examination was performed by a trained physician using standardized diagnostic criteria. Acanthosis nigricans was defined as hyperpigmented, velvety thickening of the skin, particularly over the neck and axillae; diabetic dermopathy as round or oval, atrophic brown macules on the anterior shins; and necrobiosis lipoidica as well-demarcated, yellow-brown plaques with telangiectasia. All dermatologic assessments were performed by the same examiner to minimize inter-observer bias.

For the assessment of NAFLD and GI complications, all patients underwent abdominal ultrasonography performed by a single experienced radiologist using standardized grading criteria for hepatic steatosis based on hepatic echogenicity relative to the kidney cortex. Relevant laboratory investigations, including liver function tests, serum lipid profile, and fasting blood glucose, were also obtained. GI symptoms such as nausea, vomiting, bloating, constipation, and diarrhea were evaluated using a structured symptom scoring checklist developed in consultation with gastroenterologists. Each symptom was rated according to its frequency and severity based on patient interviews and clinical examination. The checklist was pretested on a small local sample to ensure clarity, internal consistency, and suitability for the study population.

Data analysis

All data were entered and analyzed using SPSS Statistics, version 26.0 (IBM Corp., Armonk, NY). The Shapiro-Wilk test was applied to assess the normality of continuous variables. Continuous variables were expressed as mean ± standard deviation, while categorical variables were presented as frequencies and percentages. The chi-square test was used to evaluate the relationship between cutaneous findings and NAFLD or other GI complications.

Multivariate logistic regression analysis was performed to control for potential confounders, including age, gender, body mass index (BMI), HbA1c, and duration of diabetes. Multicollinearity among independent variables was tested using variance inflation factors (VIF < 5 considered acceptable), and model fit was assessed using the Hosmer-Lemeshow goodness-of-fit test and Nagelkerke R². Statistical significance was defined as p < 0.05.

Ethical considerations

Before the study started, the LRH Institutional Review Board (IRB) granted ethical approval (Ref. No. 923, Dated: 21.7.2023). Following an explanation of the study’s goals and methods, each participant provided signed informed consent.

## Results

The study included 328 diabetic patients, whose mean age was 52.4 ± 11.8 years (range: 18-78 years). There were 144 (43.9%) females and 184 (56.1%) males among them. As shown in Table 1, the majority had type 2 diabetes (n = 263; 80.2%), while 65 (19.8%) were diagnosed with type 1 diabetes. The mean HbA1c level was 8.3 ± 1.6%, indicating inadequate glycemic control; notably, 238 (72.6%) patients had HbA1c ≥ 7%, reflecting a high prevalence of uncontrolled diabetes in this cohort. The mean duration of diabetes was 8.7 ± 5.4 years, and the mean BMI was 28.6 ± 4.9 kg/m², indicating that most participants were overweight or obese. These baseline clinical and demographic characteristics offer crucial background information for analyzing the correlations and incidence of cutaneous symptoms, NAFLD, and digestive issues among the participants in the study.

**Table 1:** Baseline demographic and clinical characteristics Categorical variables are shown as n (%) and continuous variables as mean ± SD. Uncontrolled glycemia (HbA1c ≥ 7%) was observed in 238 (72.6%) patients, indicating suboptimal diabetes control in the majority of the study population. BMI, body mass index

Variable	Mean ± SD, n (%)
Total patients	328
Age (years)	52.4 ± 11.8
Gender	
Male	184 (56.1%)
Female	144 (43.9%)
Duration of diabetes (years)	8.7 ± 5.4
HbA1c (%)	8.3 ± 1.6
BMI (kg/m²)	28.6 ± 4.9
Type of diabetes	
Type 1	65 (19.8%)
Type 2	263 (80.2%)

**Figure 1:**
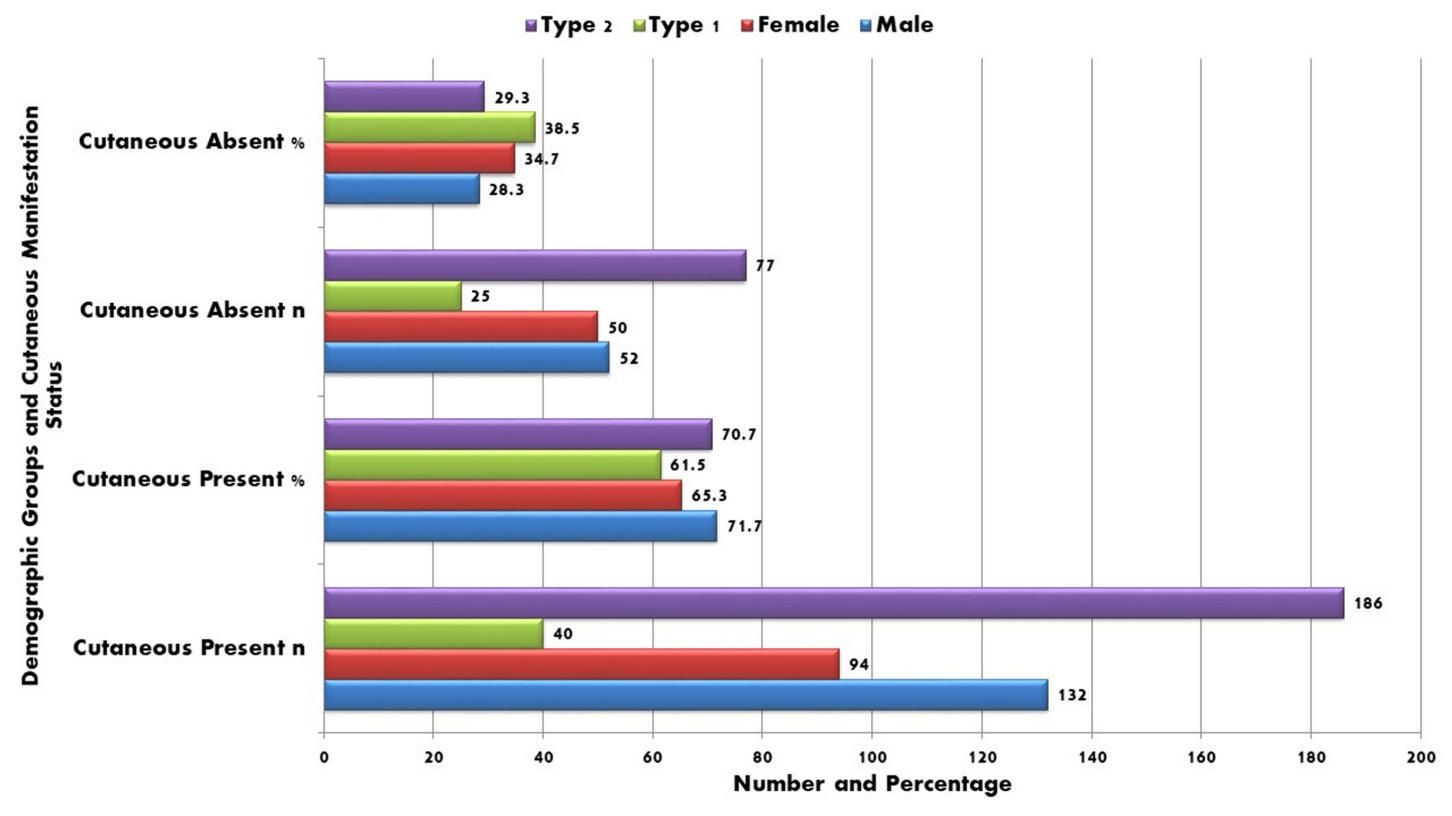
Cutaneous manifestations by gender and diabetes type The chi-square test was applied for gender and diabetes type comparisons; a p-value of <0.05 was considered statistically significant.

Figure 1 shows that 71.7% (n = 132) of males and 65.3% (n = 94) of females experienced cutaneous manifestations, with no statistically significant difference between genders. Likewise, skin abnormalities were seen in 40 (61.5%) of 65 patients with type 1 diabetes and 186 (70.7%) of 263 patients with type 2 diabetes; this difference was likewise not statistically significant (χ² = 2.01, p = 0.16). The prevalence of cutaneous manifestations was similar for both genders and diabetes types, according to these findings, indicating that demographic factors did not significantly affect dermatological involvement in this study sample.

**Figure 2:**
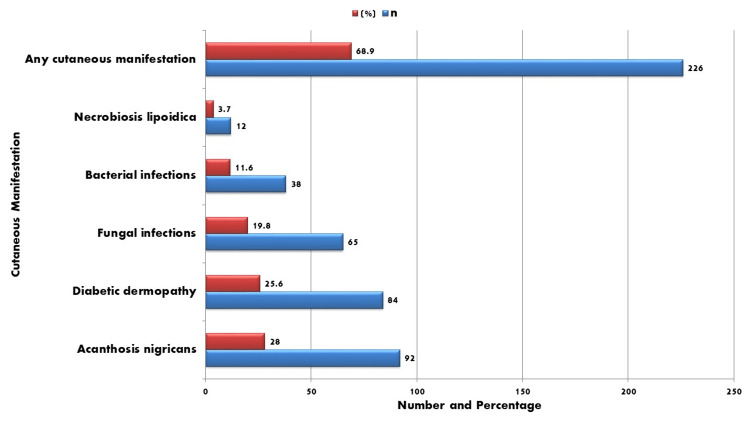
Prevalence of cutaneous manifestations Data are presented as n (%). No statistical test was applied.

Cutaneous manifestations were observed in 226 (68.9%) of the 328 participants. As illustrated in Figure 2, the most frequently reported skin changes were acanthosis nigricans in 92 (28.0%) patients and diabetic dermopathy in 84 (25.6%) patients. Fungal infections occurred in 65 (19.8%) participants, while bacterial infections were noted in 38 (11.6%). Necrobiosis lipoidica was relatively rare, affecting 12 (3.7%) patients. These results emphasize the value of a dermatological examination in diabetics since early detection of skin symptoms may help identify patients who are susceptible to systemic problems.

Using ultrasonography, 138 (42.1%) of the 328 patients were diagnosed with NAFLD, as shown in Table 2. NAFLD was present in 108 (47.8%) of the patients with cutaneous signs and 30 (28.3%) of the individuals without skin abnormalities. This corresponds to a crude OR of 2.29 (95% CI: 1.38-3.81), indicating that diabetic patients with cutaneous manifestations had more than twice the odds of developing NAFLD compared to those without such findings. The association was statistically significant (χ² = 10.8, p = 0.001), suggesting that dermatological findings may serve as useful clinical indicators of hepatic involvement in diabetic individuals.

**Table 2:** Association of cutaneous manifestations with NAFLD The chi-square test was applied; Fisher’s exact test was used where appropriate. Crude ORs with 95% CI are presented. A p-value of <0.05 was considered statistically significant. NAFLD was significantly more prevalent among patients with cutaneous manifestations, obesity (BMI ≥ 30 kg/m²), poor glycemic control (HbA1c ≥ 8%), and longer diabetes duration (>10 years), whereas gender and age were not significantly associated. NAFLD, non-alcoholic fatty liver disease; BMI, body mass index

Variable	NAFLD present, n (%)	NAFLD absent, n (%)	Total	Crude OR (95% CI)	Statistical test	x^2^-value	p-value
Cutaneous manifestation	108 (47.8%)	118 (52.2%)	226	2.29 (1.38-3.81)	Chi-square	10.8	0.001
Absent	30 (28.3%)	72 (71.7%)	102	-	-	-	-
BMI ≥ 30 kg/m²	84 (60.9%)	54 (39.1%)	138	3.26 (2.00-5.31)	Chi-square	25.4	<0.001
BMI < 30 kg/m²	54 (28.4%)	136 (71.6%)	190	-	-	-	-
HbA1c ≥ 8%	94 (47.2%)	105 (52.8%)	199	1.85 (1.14-3.00)	Chi-square	6.55	0.01
HbA1c < 8%	44 (32.0%)	93 (68.0%)	137	-	-	-	-
Duration of diabetes >10 years	72 (52.2%)	66 (47.8%)	138	1.91 (1.18-3.10)	Chi-square	7.01	0.008
≤10 years	66 (33.7%)	130 (66.3%)	196	-	-	-	-
Age ≥ 60 years	54 (44.3%)	68 (55.7%)	122	1.13 (0.72-1.77)	Chi-square	0.32	0.57
<60 years	84 (41.8%)	117 (58.2%)	201	-	-	-	-
Male gender	80 (43.5%)	104 (56.5%)	184	1.03 (0.66-1.61)	Chi-square	0.02	0.89
Female gender	58 (41.0%)	83 (59.0%)	141	-	-	-	-

GI symptoms were assessed in all participants. As shown in Table 3, among patients with cutaneous manifestations, constipation was reported in 72 (31.9%) and bloating in 61 (27.0%), both significantly higher than in patients without skin changes, who experienced constipation in 20 (19.6%) and bloating in 17 (16.7%) (χ² = 5.45, p = 0.02 and χ² = 4.14, p = 0.04, respectively). In contrast, diarrhea and nausea/vomiting were not significantly associated with dermatological findings, occurring in 30 (13.3%) versus 12 (11.8%) and 25 (11.1%) versus 10 (9.8%) of patients with and without cutaneous manifestations, respectively (p > 0.05). According to these findings, diabetes patients who exhibit skin signs may be more likely to experience specific GI issues, namely bloating and constipation.

**Table 3:** Association between cutaneous manifestations and gastrointestinal symptoms The chi-square test was applied for constipation, bloating, diarrhea; Fisher’s exact test was applied for nausea/vomiting. A p-value of <0.05 is statistically significant. GI, gastrointestinal

GI symptom	Cutaneous present, n (%)	Cutaneous absent, n (%)	Statistical test	Value	p-value	Significance
Constipation	72 (31.9%)	20 (19.6%)	Chi-square	5.45	0.02	Significant
Bloating	61 (27.0%)	17 (16.7%)	Chi-square	4.14	0.04	Significant
Diarrhea	30 (13.3%)	12 (11.8%)	Chi-square	0.13	0.70	Not significant
Nausea/vomiting	25 (11.1%)	10 (9.8%)	Fisher’s exact	0.09	0.71	Not significant

To find independent predictors of NAFLD while controlling for relevant confounders such as age, gender, BMI, length of diabetes, and HbA1c, multivariate logistic regression analysis was used. As shown in Table 4, cutaneous manifestations were associated significantly with NAFLD, with an OR of 2.05 (95% CI: 1.25-3.35, p = 0.004). Obesity (BMI ≥ 30 kg/m²) emerged as a strong predictor (OR = 3.12, 95% CI: 1.88-5.19, p < 0.001), and poor glycemic control (HbA1c ≥ 8%) was also significantly associated with NAFLD (OR = 1.67, 95% CI: 1.02-2.74, p = 0.04). In contrast, duration of diabetes >10 years (OR = 1.54, 95% CI: 0.94-2.54, p = 0.08), age ≥ 60 years (OR = 1.22, 95% CI: 0.76-1.96, p = 0.41), and male gender (OR = 1.18, 95% CI: 0.73-1.90, p = 0.50) were not significant predictors. These findings suggest that poor glycemic control, obesity, and dermatological symptoms all independently raise the risk of NAFLD in diabetic patients.

**Table 4:** Multivariate logistic regression for predictors of NAFLD Multivariate logistic regression adjusted for potential confounders (age, gender, BMI, HbA1c, and duration of diabetes). *A p-value of <0.05 is considered statistically significant. Model fit: Nagelkerke R² = 0.29; Hosmer–Lemeshow χ² = 6.84, p = 0.55, indicating good calibration and moderate explanatory power. NAFLD, non-alcoholic fatty liver disease; BMI, body mass index

Variable	OR	95% CI	p-value*
Cutaneous manifestation	2.05	1.25-3.35	0.004
BMI ≥ 30 kg/m²	3.12	1.88-5.19	<0.001
Duration of diabetes > 10 years	1.54	0.94-2.54	0.08
HbA1c ≥ 8%	1.67	1.02-2.74	0.04
Age ≥ 60 years	1.22	0.76-1.96	0.41
Male gender	1.18	0.73-1.90	0.50

## Discussion

This study underscores the close interrelationship between dermatological, hepatic, and GI manifestations in diabetic patients, highlighting the skin as a visible marker of systemic metabolic dysfunction. The observed burden of cutaneous disorders in our sample (68.9%) aligns with previous regional data showing that skin manifestations are markedly more frequent among diabetics compared with non-diabetic individuals, in whom prevalence rates typically range between 20% and 25% across South Asian populations [19]. This reinforces that dermatologic findings can reflect underlying metabolic imbalance and microvascular injury associated with chronic hyperglycemia.

The occurrence of NAFLD in 42.1% of our cohort reflects the growing recognition of hepatic involvement as a metabolic complication of diabetes in Pakistan. These findings suggest that early recognition of cutaneous changes may aid in identifying patients at higher risk for NAFLD and GI disturbances, emphasizing the need for an integrated diagnostic approach. Multivariate logistic regression identified cutaneous manifestations, BMI ≥ 30 kg/m², and HbA1c ≥ 8% as independent predictors of NAFLD, while gender, age, and diabetes duration were not significant predictors. The lack of gender- or diabetes-type-based differences in dermatological findings further supports the notion that metabolic control and obesity, rather than demographic factors, drive these associations.

The prevalence pattern of skin manifestations in this study aligns with prior literature, confirming that acanthosis nigricans and diabetic dermopathy are the most frequent dermatologic findings among diabetic patients [19,20]. These conditions represent clinical expressions of insulin resistance and chronic hyperglycemia, consistent with established pathogenic mechanisms. Similarly, the observed prevalence of NAFLD corresponds to existing evidence indicating that a substantial proportion of diabetic patients develop fatty liver independent of alcohol intake [21].

The significant association between cutaneous manifestations and NAFLD highlights a potential shared metabolic basis, particularly insulin resistance, obesity, and oxidative stress [22]. Patients with elevated BMI and poor glycemic control demonstrated a higher risk of NAFLD, reinforcing the metabolic continuum between skin and liver involvement [23]. This finding supports the use of dermatological examination as a non-invasive, early clinical indicator of systemic metabolic dysfunction.

The increased frequency of GI symptoms, particularly constipation and bloating, among patients with skin manifestations further suggests that autonomic neuropathy and metabolic dysregulation may concurrently affect multiple organ systems in diabetes. Although diarrhea and nausea/vomiting were not significantly associated, the results imply that certain GI symptoms, likely linked to autonomic and motility disturbances, are more prevalent among those exhibiting visible dermatologic alterations [24].

Compared to previous studies, the independent predictive value of cutaneous manifestations for NAFLD represents an important clinical insight, suggesting that dermatological evaluation could complement hepatic and metabolic screening in diabetic patients [22-25]. By integrating dermatologic, hepatic, and GI dimensions, this study demonstrates the multisystemic nature of diabetes and underscores the importance of comprehensive, cross-disciplinary assessment [26].

Limitations and future directions

The findings of this study should be interpreted in light of certain limitations. First, as a single-center, cross-sectional study, generalizability is limited, and causal relationships cannot be established. Second, although multivariate analysis is adjusted for major confounders, uncontrolled variables such as dietary habits, physical activity, medication use, and coexisting metabolic disorders (e.g., hypertension, dyslipidemia) may have influenced the observed associations. Third, GI symptoms were assessed using a self-developed structured checklist tailored for local use rather than a validated international scale. While this tool was pretested for internal consistency, it may limit comparability with other datasets. Fourth, NAFLD diagnosis was based on ultrasonography, which, although practical and widely available, has lower sensitivity for detecting steatosis and fibrosis compared with histological or elastographic techniques.

Future research should address these gaps by employing standardized and validated symptom assessment tools and adopting advanced diagnostic modalities such as FibroScan or MRI-based imaging to enhance diagnostic accuracy and fibrosis quantification. Moreover, multicenter, longitudinal studies incorporating detailed evaluations of lifestyle factors, medication exposure, and metabolic comorbidities are warranted to confirm and extend these findings. Prospective studies exploring the temporal sequence of dermatological, GI, and hepatic complications may also clarify causal pathways and assess whether improved glycemic and weight management can mitigate both systemic and cutaneous outcomes. Such integrative work would refine early screening strategies and promote holistic care in diabetic populations.

## Conclusions

This study found that skin changes are highly common among people with diabetes and are closely linked with fatty liver disease and digestive problems such as constipation and bloating. Conditions like acanthosis nigricans and diabetic dermopathy were observed more frequently in those with poor blood sugar control and metabolic imbalance. These visible skin changes may serve as simple, non-invasive clues to identify patients at higher risk for internal complications. Even after accounting for factors such as age, gender, BMI, and glucose levels, the association between skin findings and fatty liver remained significant, highlighting their potential value in early detection of metabolic dysfunction. While the results cannot establish a direct cause-and-effect relationship, they suggest that dermatological evaluation could be an important part of diabetes care. Routine skin assessments may help identify high-risk individuals early and allow for timely dietary, lifestyle, and medical interventions to prevent further complications. Larger, long-term studies from multiple centers are needed to confirm these findings and determine how best to integrate skin examination into comprehensive diabetes management programs aimed at improving patient outcomes.

## Appendices

**Table 5:** Structured proforma for data collection (evaluation of cutaneous manifestations in patients with diabetes mellitus: association with NAFLD and other GI complications) Use one form per participant. For checkbox/choice questions, mark ✓ or give the requested value. For GI symptoms, rate frequency (0 = none, 1 = occasional, 2 = frequent, 3 = persistent) and severity (0 = none, 1 = mild, 2 = moderate, 3 = severe). NAFLD, non-alcoholic fatty liver disease; GI, gastrointestinal

No.	Variable/question	Response (write value/tick)	Options/notes
A. Administrative & demographic information			
1	Study ID		
2	Date of interview		
3	Investigator initials		
4	Informed consent given	☐ Yes ☐ No	If no → do not enroll
5	Age (years)		
6	Sex	☐ Male ☐ Female ☐ Other	
7	Residence		City/rural
8	Contact/phone (optional)		
B. Diabetes profile & comorbidities			
9	Type of diabetes	☐ Type 1 ☐ Type 2 ☐ Other	
10	Duration of diabetes (years)		
11	Current diabetes medications		List (e.g., metformin, insulin - dose/frequency)
12	Other medications (not DM)		e.g., statin, antihypertensive
13	Relevant comorbidities	☐ Hypertension ☐ IHD ☐ CKD ☐ Dyslipidemia ☐ Others: __	Specify
14	Smoking status	☐ Never ☐ Former ☐ Current	Pack-years if available
15	Alcohol history	☐ None ☐ Social ☐ Regular	Note exclusions if alcohol abuse
16	Height (cm)		
17	Weight (kg)		
18	BMI (kg/m²)		Compute = weight/(height/100)²
C. Laboratory investigations & ultrasound findings			
19	Latest HbA1c (%)		Date of sample: //__
20	Fasting blood glucose (mg/dL)		Date: //__
21	Liver function tests (report)	ALT: __ AST: __ ALP: __ Bilirubin: __	Units
22	Lipid profile (report)	Total Chol: __ LDL: __ HDL: __ TG: __	Units
23	Abdominal ultrasound for NAFLD	☐ Performed ☐ Not performed	If performed: ☐ NAFLD present ☐ absent; grade (I-III): __
24	History of known liver disease (other than NAFLD)	☐ Yes ☐ No	Specify
25	Any history of alcohol abuse	☐ Yes ☐ No	If yes → exclude per protocol
D. Dermatological assessment			
26	Dermatological examination performed by (initials)		
27	Cutaneous manifestations (tick all that apply)	☐ Acanthosis nigricans ☐ Diabetic dermopathy ☐ Necrobiosis lipoidica ☐ Fungal infection ☐ Bacterial infection ☐ Other: __	If other → describe
28	Onset/duration of skin finding(s)		e.g., months/years
29	Was the lesion biopsied or referred?	☐ Yes ☐ No	Details: __
E. Gastrointestinal assessment (symptom checklist)			
30	Nausea	Frequency: __ /severity: __	Frequency 0-3; severity 0-3
31	Vomiting	Frequency: __ /severity: __	
32	Bloating	Frequency: __ /severity: __	
33	Constipation	Frequency: __ /severity: __	
34	Diarrhea	Frequency: __ /severity: __	
35	Other GI symptoms (specify)		e.g., early satiety, abdominal pain; rate 0-3
36	Total GI frequency score		Sum of frequency items (30-35)
37	Total GI severity score		Sum of severity items (30-35)
38	Any recent GI investigations (endoscopy, CT)	☐ Yes ☐ No	Date & findings: __
39	Hospitalizations related to GI or liver disease (last 12 months)	☐ Yes ☐ No	If yes: date/reason
F. Treatment & preventive history			
40	Any skin-related treatment in the last 3 months	☐ Topical ☐ Systemic ☐ None	Specify drug
41	Vaccination status (HBV)	☐ Vaccinated ☐ Not vaccinated ☐ Unknown	Date if vaccinated
42	Pregnancy/breastfeeding status	☐ Pregnant ☐ Breastfeeding ☐ Neither	Exclude if pregnant
G. Administrative completion			
43	Laboratory/sample collection dates		List: HbA1c __ LFT __ Lipids __ US __
44	Notes/clinician comments		
45	Form completed by (name & signature)		
